# Interactions in the 2×2×2 factorial randomised clinical STEPCARE trial and the potential effects on conclusions: a protocol for a simulation study

**DOI:** 10.1186/s13063-022-06796-7

**Published:** 2022-10-22

**Authors:** Markus Harboe Olsen, Aksel Karl Georg Jensen, Josef Dankiewicz, Markus B. Skrifvars, Matti Reinikainen, Marjaana Tiainen, Manoj Saxena, Anders Aneman, Christian Gluud, Susann Ullén, Niklas Nielsen, Janus Christian Jakobsen

**Affiliations:** 1 Copenhagen Trial Unit, Centre for Clinical Intervention Research, The Capital Region, Copenhagen University Hospital, Rigshospitalet, Copenhagen, Denmark; 2 Department of Neuroanaesthesiology, The Neuroscience Centre, Copenhagen University Hospital, Rigshospitalet, Copenhagen, Denmark; 3 Section of Biostatistics, Department of Public Health, University of Copenhagen, Copenhagen, Denmark; 4 Department of Clinical Sciences, Cardiology, Lund University, Skåne University Hospital Lund, Lund, Sweden; 5 Department of Emergency Care and Services, Helsinki University Hospital and University of Helsinki, Helsinki, Finland; 6 Department of Anaesthesiology and Intensive Care, Kuopio University Hospital and University of Eastern Finland, Kuopio, Finland; 7 Department of Anesthesiology, Intensive Care, and Pain Medicine, Helsinki University and Helsinki University Hospital, Helsinki, Finland; 8 Critical Care Division, the George Institute for Global Health, University of New South Wales, Sydney, Australia; 9 Intensive Care Unit, Liverpool Hospital, South Western Sydney Local Health District, Sydney, Australia; 10 South Western Clinical School, University of New South Wales, Sydney, Australia; 11 Department of Regional Health Research, The Faculty of Health Sciences, University of Southern Denmark, Odense, Denmark; 12 Clinical Studies Sweden – Forum South, Skåne University Hospital, Lund, Sweden; 13 Department of Clinical Sciences Lund, Anesthesiology and Intensive Care, Lund University, Helsingborg Hospital, Helsingborg, Sweden

**Keywords:** Simulation study, Protocol, Interactions, Factorial design, Randomised clinical trial, Statistical analysis plan

## Abstract

**Background:**

Randomised clinical trials with a factorial design may assess the effects of multiple interventions in the same population. Factorial trials are carried out under the assumption that the trial interventions have no interactions on outcomes. Here, we present a protocol for a simulation study investigating the consequences of different levels of interactions between the trial interventions on outcomes for the future 2×2×2 factorial designed randomised clinical Sedation, TEmperature, and Pressure after Cardiac Arrest and REsuscitation (STEPCARE) trial in comatose patients after out-of-hospital cardiac arrest.

**Methods:**

By simulating a multisite trial with 50 sites and 3278 participants, and a presumed six-month all-cause mortality of 60% in the control population, we will investigate the validity of the trial results with different levels of interaction effects on the outcome. The primary simulation outcome of the study is the risks of type-1 and type-2 errors in the simulated scenarios, i.e. at what level of interaction is the desired alpha and beta level exceeded. When keeping the overall risk of type-1 errors ≤ 5% and the risk of type-2 errors ≤ 10%, we will quantify the maximum interaction effect we can accept if the planned sample size is increased by 5% to take into account possible interaction between the trial interventions. Secondly, we will assess how interaction effects influence the minimal detectable difference we may confirm or reject to take into account 5% (small interaction effect), 10% (moderate), or 15% (large) positive interactions in simulations with no ‘true’ intervention effect (type-1 errors) and small (5%), moderate (10%), or large negative interactions (15%) in simulations with ‘true’ intervention effects (type-2 errors). Moreover, we will investigate how much the sample size must be increased to account for a small, moderate, or large interaction effects.

**Discussion:**

This protocol for a simulation study will inform the design of a 2×2×2 factorial randomised clinical trial of how potential interactions between the assessed interventions might affect conclusions. Protocolising this simulation study is important to ensure valid and unbiased results.

**Trial registration:**

Not relevant

**Supplementary Information:**

The online version contains supplementary material available at 10.1186/s13063-022-06796-7.

## Introduction

Randomised clinical trials most often investigate the effects of a single intervention [1]. Randomised clinical trials with a factorial design may assess the effects of multiple interventions on outcomes in the same population. The outcome relating to the effect of each of the assessed interventions included in the factorial design is often reported in separate articles under the assumption that there are no interactions between the effects of the trial interventions [2]. However, interaction between the assessed interventions included in the factorial design may influence the overall trial results [2].

The Sedation, TEmperature, and Pressure after Cardiac Arrest and REsuscitation (STEPCARE) trial is a large phase 3 investigator-initiated, international, assessor-blinded, randomised 2×2×2 factorial trial. We plan to assess the following hypotheses in 3278 adult patients who are comatose after resuscitation from out-of-hospital cardiac arrest (OHCA) (population): does a deep sedation target, the use of a feedback-directed device to target normothermia (< 37.8°C), or a mean arterial pressure (MAP) target > 85 mmHg (the interventions 1, 2, and 3) compared with sedation minimisation, temperature management without a device, or a target MAP of > 65 mmHg (the comparators 1, 2, and 3) improve survival at 6 months after randomisation (the primary clinical outcome)?

We plan to publish the results of these three interventions separately, under the assumption of no significant between-intervention interactions affecting survival at 6 months. We anticipate that the interventions may have a short-term impact on each other (‘physiological interaction’). In previous studies in critical care populations studying the relationship between short-term changes in physiology and longer-term patient-centred outcomes, the associations have been inconsistent and unpredictable [3–5]. As examples, the intensive control of blood sugar to normal levels in a general intensive care population was associated with both increased and reduced mortality [3, 5]; and the substantial lowering of intracranial pressure after traumatic brain injury by surgical intervention was associated with worse outcomes [4]. In addition, in our previous trial with partial co-recruitment in a 2×2 factorial randomisation with the Targeted Mild Hypercapnia after Cardiac Arrest (TAME) trial, there was no observed interaction between the interventions affecting outcomes [6, 7]. Likewise, we found no significant interactions affecting patient outcomes between PaCO_2_, arterial oxygen tension, MAP, or temperature targets in the Carbon dioxide, Oxygen, and Mean Arterial pressure after Cardiac Arrest and REsuscitation (COMACARE) trial [8, 9]. However, it is theoretically possible that there are physiological interactions between our three experimental interventions, and such interactions may influence the outcome of the participants. The actual levels of such physiological interaction and their relationship to outcome for these specific interventions are, however, unknown.

In the preparation of the STEPCARE trial, we will therefore carry out a simulation study investigating the consequences of different levels of interactions between the trial interventions in relation to the primary clinical outcome. We aim to investigate the type-1 error with increasing positive interaction effects in simulations with no ‘true’ intervention effect and the type-2 error with increasing negative interaction effects in simulations with ‘true’ intervention effects. Simulation studies are seldom protocolised [10], but since simulations may be just as influenced by data driven approaches as other types of analyses, we will in detail define our methodology before we run the simulations [11–13].

## Methods

This simulation study will investigate how interactions between trial interventions in a 2×2×2 factorial design randomised clinical trial potentially influence the overall results. The planned STEPCARE randomised clinical trial will include patients after OHCA, and the simulations are based on information gained from two previous trials investigating targeted temperature management in patients with OHCA [6, 14]. In brief, the first trial randomised 950 participants after OHCA to a temperature of 33 °C versus 36 °C during the first 36 h after randomisation. The second trial randomised 1900 participants after OHCA to a temperature of 33 °C versus normothermia (≤ 37.8 °C) during the first 40 h after randomisation. Both trials concluded that hypothermia did not influence the risk of either death or poor functional outcome at 6 months [6, 14].

### Study design

The simulated randomised clinical trial will be a 2×2×2 factorial design, where each participant will be randomly assigned to the experimental versus the control group for each of the trial interventions (temperature control with the use of a feedback-directed device versus no device for temperature control; protocolised sedation versus sedation minimisation; higher MAP target versus usual MAP target). The primary clinical outcome will be 6-month all-cause mortality in all simulations. Randomisation will be stratified according to trial site with varying block sizes. The different situations will be investigated with 1000 simulations for each condition, which is sufficient to achieve a stable result [15–17]. The data generating mechanism is available as Supplemental Material.

### Simulation

The sRCT-function, which is implemented into the publicly available *clintools*-package for R, has been developed to simulate randomised clinical trials [18]. The sRCT-function generates an individual participation table with site, allocation, relative risks, and outcome. This table is generated through these steps:

- The sites are created, and the probability of a participant being included in a site is defined. To simulate that each site does not include the same number of participants, the probability of a participant being included in the specific site is based on a randomly generated truncated normal distributed probability (the shape of the distribution: mean 10, SD 5). Each site receives a number and by dividing the sum of the numbers allocated to all sites with the specific site, the probability of inclusion is identified. The probabilities will be recalculated until all sites have a probability of including more than 0.5% of the participants. This means that the smallest site in a multisite trial with 50 sites and 3278 participants would include 16 (median, interquartile range (IQR) 11 to 20) participants and the largest would include 135 (median, IQR 125 to 148) participants.
- An allocation table is generated for each site based on the defined block sizes and participants in each site are included in the trial and allocated to each intervention.
- The baseline risk of the outcome is randomly assigned to each site with a low variability (standard deviation of 0.05), to simulate a random effect of site. The baseline risk is for this trial defined as 60% (*see below)*.
- The outcome probability (absolute risk) for each participant is calculated based on the following formula:
  $$\ln \left(P\left(Y=1\right)\right)={a}_0+{Var}_1\bullet {a}_1+{Var}_2\bullet {a}_2+{Var}_3\bullet {a}_3+{Var}_1\bullet {Var}_2\bullet {a}_4\dots$$

α_0_ refers to the natural logarithm of the probability of the outcome. α_1-3_ refers to the natural logarithm of the relative risk reduction or increase for interventions. α_4+_ refers to the natural logarithm of the relative risk reduction or increase for interactions. Var_x_ is 0 if the participant is in the control arm and 1 if the participant is in the intervention arm.

- The outcome is calculated for each participant in each simulation based on the outcome probability calculated above.

### Simulated study population and relative risk reductions

The simulated trials will be carried out in a multisite setup with an average of 50 participating sites (SD 5), and participants will have a presumed 6-month all-cause mortality of 60% in the control group [6, 14]. Based on the sample size of 3278 participants, the alpha for each intervention set at 0.05, and the minimal important absolute risk reduction at 5.6% (corresponding to a 9.3% relative risk reduction, RRR), we would achieve a power of 90% in each comparison. To investigate the influence of sample size, we will carry out all investigating using two smaller samples sizes. The first includes 1,990 participants and assumes a minimal important absolute risk reduction of 7.2% (corresponding to a 12% RRR), which results in a power of 90%. The second includes 200 participants and assumes an absolute risk reduction of 22.7% (corresponding to a 37.8%), which results in a power of 90%. Missing data will not be included in the simulations.

### Statistical analysis of the simulated trials

The simulated trials will have a dichotomous primary simulation outcome and the effect of interaction of one intervention will be analysed, referred to as ‘*the evaluated intervention*’. Relative risks (RRs) and the corresponding confidence intervals will be derived by the glmer function with binomial(log) as family from the lme4 package [19], where a *p* value less than 0.05 will be considered significant [20]. If the model does not converge, we will calculate using the glm-function from base R with family set as *qausipoisson* (Supplemental Material). We will test for interaction between interventions. Interaction will be tested between the evaluated intervention and the two other interventions in this simulated study. We will only consider that there is evidence of an interaction if the interaction is statistically significant with a *p*-value below 0.017 (Bonferroni-corrected per number of possible two-way interactions for each analysis with an original alpha of 0.05) [21].

### Interaction effects

Based on theoretical considerations, we believe that an interaction effect of 5% (small interaction effect), 10% (moderate interaction effect), or 15% (the largest plausible interaction effect) of the presumed effect size from the sample size calculation would be the most plausible. We used the following formula to ascertain the actual interaction in percentage of the most plausible interaction effects:

$$iRR= RRR-\left(1-{e}^{\ln \left(1- RRR\right)+\ln \left(1-x\right)}\right)$$

*RRR* is the presumed relative risk reduction from the sample size analysis, *iRR* is the relative risk of the interaction (interaction effect), and *x* is the percentage of interaction. Based on the formula above, and after isolation of *x*:

$$x=\frac{iRR}{1- RRR}$$

For the primary sample size of 3278 with a presumed effect size of 9.3% (*RRR*), a small interaction effect of 5% would correspond to a relative risk reduction or increase of 0.465% (*iRR*), and the interaction (*x*) in percentage is 0.513%. See Table 1 for all most plausible interaction effects.

**Table 1:** Interaction effects

	Interaction effects		
	Small	Moderate	Large
**Presumed relative risk reduction (9.3%)**	**9.3%**	**9.3%**	**9.3%**
**Percentage interaction of the presumed relative risk reduction**	**5%**	**10%**	**15%**
**Relative risk reduction for the interaction**	**0.465%**	**0.93%**	**1.395%**
**Interaction**	**0.513%**	**1.025%**	**1.538%**

### Simulated conditions

This will be assessed using the following conditions:

1. Increasing positive interaction (i.e. a synergistic effect) between the evaluated intervention and one other intervention.
2. Increasing positive interaction between the evaluated intervention and the other two interventions.
3. Increasing negative interaction between the evaluated intervention and one other intervention.
4. Increasing negative interaction between the evaluated intervention and the other two interventions.

For each of the four conditions we will investigate two effective (RRR from the sample size calculation and double of that, e.g. for primary analysis RRR of 0.093 and RRR of 0.186) interventions and two minimally harmful (RR: 1.001 and 1.0025) interventions.

### Outcomes

**Fig. 1:**
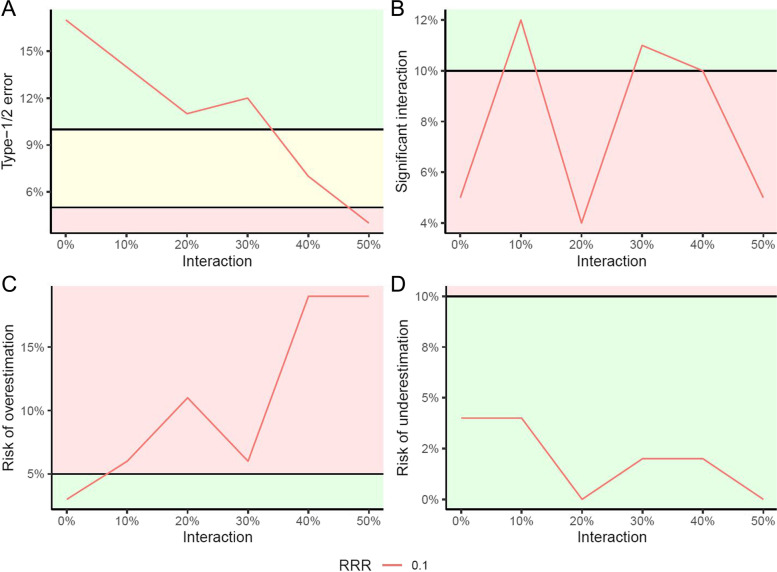
This figure present one condition with one effective intervention (relative risk reduction (RRR) of 0.1) and a positive interaction from one other intervention. This pre-emptive simulation only includes simulation of 10% increments, 100 simulated trials, and a G-computation of the relative risk with 100 iterations. **A** The primary simulation outcome is evaluated based on the combined risk of type-1 and type-2 errors. For effective interventions the threshold of 10% (*y*-axis) is used to evaluate at what degree of interaction the risk of type-2 error is above the defined 10%, while for ineffective interventions the threshold of 5% (y-axis) is used to evaluate the risk of type-1 error. **B** The secondary outcome of when significant interaction is identified is set at 10%. The exploratory outcomes of over- (**C**) and underestimations (**D**) are based on the confidence limits of RR, where the thresholds are set equally to the alpha and beta of our simulations, i.e. 5% and 10%, respectively

The primary simulation outcome of this study will assess at what level of interaction (1) the risk of concluding that an ineffective intervention is effective is higher than the acceptable 5% (the chosen alpha level); and (2) at what level of interaction the risk of concluding that an effective intervention is actually ineffective is higher than the acceptable 10% (the chosen beta level). This will inform us what degree of interaction influences the results, and when the assumption of no interaction between interventions is violated (Fig. 1A). When keeping the overall risk of type-1 errors ≤ 5% and the risk of type-2 errors ≤ 10%, we will quantify the maximum interaction effect we can accept if the planned sample size is increased by 5% to take into account possible interaction between the trial interventions. Secondly, we will assess how interaction effects influence the minimal detectable difference we may confirm or reject to take into account 5% (small interaction effect), 10% (moderate), or 15% (large) positive interaction effects in simulations with no ‘true’ intervention effect (type-1 errors) and small (5%), moderate (10%), or large (15%) negative interactions in simulations with ‘true’ intervention effects (type-2 errors). Finally, we will investigate how much the sample size must be increased to account for a small, moderate, and large interaction effects.

The exploratory outcomes include at what level of interaction the analysis of interaction will achieve significance in more than 90% of the trials (the power) [22]. This will inform us at what degree of interaction the trialist will be informed about the breach in the assumption of no interaction between interventions (Fig. 1B). Furthermore, at what level of interaction the risk of overestimation of effect surpasses the acceptable 5% (Fig. 1C), at what level of interaction the risk of underestimation of effect surpasses the acceptable 10% (Fig. 1D), and the difference between the average calculated relative risk and the actual intervention effect. In trials designed with an alpha of 5% and a power of 90%, a false positive (type-1 error) rate of 5% and a false negative (type-2 error) rate of 10% is accepted. Type-1 and type-2 errors refer to the H_0_ with the assumption that interventions can only be inferior, superior, or equal [23]. A more granular interpretation of a type-1 error is an overestimation of an actual intervention effect, while a type-2 error is an underestimation of an actual intervention effect. Overestimation proportion is calculated by identifying the number of simulated trials where the lower confidence limit of RR is above the actual RR, and underestimation proportion is calculated by identifying the number of simulated trials where the upper confidence limit is below the actual RR.

## Results

We ran the following simulations to test the validity of the sRCT-function. The sRCT-function on average results in an RR of 0.90 (95% confidence interval (CI) 0.82 to 0.95) when running 10,000 simulations with trials of pragmatically chosen 2000 participants, an intervention of 10% RRR, and no interactions. Running 18,000 simulations with varying trial sizes (median 3,588; IQR: 300–3850 participants), varying intervention effects (6000 simulations with RRR of 0.0; 6000 simulations with RRR of 0.1; and 6,000 simulations with RRR of 0.2), and no interaction, we found 4.98% of the trials found a confidence interval not including the actual interventional effect. Furthermore, no confidence intervals calculated using Wald’s approximation overlapped 1.0 with a significant *p* value, and no confidence intervals did not include 1.0 with an insignificant *p* value. Unfortunately, 4216 of our analyses did not converge; thus, site was then included as a fixed effect in these analyses. We believe that these results validate the sRCT-function. Each condition will be presented in a figure (Fig. 1).

## Discussion

Here we present a detailed protocol and statistical analysis plan for a simulation study of a factorial randomised clinical trial. This study will investigate the risks of type-1 and type-2 errors based on the level of potential interactions for our planned STEPCARE 2×2×2 factorial randomised clinical trial, including how much the sample size should be increased to take into account plausible interaction effects. Previously, a simulation study found that positive interaction between interventions affects the conclusions [24]. However, this study did not investigate at what degree of interaction the risk of type-1 and type-2 errors exceeds the decided alpha and beta nor when the investigators would be made aware of the issue. The relative risk reductions investigated in this simulation study are clinically meaningful, as well as it can be argued that even smaller intervention effects can classified as clinically meaningful [6].

### Strengths

This is the first peer-reviewed publication of a protocol and statistical analysis plan for a simulation study of a 2×2×2 factorially designed trial. Furthermore, simulation studies are rarely protocolised with pre-published peer reviewed protocols [10]. Simulation studies make an important contribution to the scientific community and are just as susceptible to data driven analyses and publication bias [25]. This protocol entails definition of investigative methods, definitive outcomes, and is accompanied by the source-code. The results from this simulation study are relevant for the design of any future randomised clinical trials with factorial designs. This protocol has been developed using recommendations for statistical analysis plans for randomised clinical trials and observational studies [11, 12].

### Limitations

Simulation studies are limited by trying to mimic real life. Therefore, simulation studies are limited by the choices in the code, but the use of previously collected aggregated measures from real-life participant data may increase the validity and generalisability of the results of the present study. Furthermore, simulation studies might not be translatable. The results of a simulation study need to be interpreted by methodologists and trialists and not implemented blindly. The size of the blocks in a factorial 2×2×2 randomised clinical trials is at minimum 8, and if the block sizes are varying the other two minimal sizes are 16 and 24. The rather large block sizes have the potential to distort the groups if smaller sites are included. This potential bias will not be investigated in the present simulation study. This simulation study will not investigate all potential aspects of interactions, and especially the influence of subgroup interventions and missing data might be important to investigate in subsequent studies.

## Conclusions

This protocol for a simulation study will inform the design of a 2×2×2 factorial randomised clinical trial, and at what level of interaction the validity of trial results would be affected. Protocolising this simulation study is important to ensure valid and unbiased results.

## Supplementary Information

**Additional file 1.**

## Data Availability

The datasets generated and/or analysed during the current study will be available at Zenodo upon publication of final study results.
