# Supplementary material for: Interactions in the 2×2×2 factorial randomised clinical STEPCARE trial and the potential effects on conclusions: a protocol for a simulation study
Source: Trials. 2022 Oct 22;23:889. doi: 10.1186/s13063-022-06796-7 (PMC9587583; doi:10.1186/s13063-022-06796-7)
Supplement: Supplementary file 1 — Additional file 1. [file 13063_2022_6796_MOESM1_ESM.docx]

Data generating mechanism

# CREATING RCT

df <- suppressMessages(suppressWarnings(try(sRCT(n_pop=part, n_sites=sites,

design = c(2,2,2), rrr=rrr,

outcome_risk = 0.60,

interaction=interaction), silent=T)))

if("try-error" %in% class(df)) next

# PRIMARY ANALYSIS

m1 <- suppressMessages(suppressWarnings(try(lme4::glmer(factor(outcome) ~ Var1 + (1|site), data = df, family=binomial(log)),silent=T)))

if("try-error" %!in% class(m1)){

res <- exp(cbind(fixef(m1), confint(m1, method = 'Wald')[-1,]))

simulations$rr[i] <- res[2,1]

simulations$lcl[i] <- res[2,2]

simulations$ucl[i] <- res[2,3]

simulations$p_val[i] <- summary(m1)$coefficients[2,4]

simulations$model[i] <- "glmer"

}else{

m1 <- suppressMessages(suppressWarnings(try(lme4::glmer(factor(outcome) ~ Var1 + (1|site), data = df, family=binomial(log), nAGQ = 0),silent=T)))

if("try-error" %!in% class(m1)){

res <- exp(cbind(fixef(m1), confint(m1, method = 'Wald')[-1,]))

simulations$rr[i] <- res[2,1]

simulations$lcl[i] <- res[2,2]

simulations$ucl[i] <- res[2,3]

simulations$p_val[i] <- summary(m1)$coefficients[2,4]

simulations$model[i] <- "glmer_nAGQ=0"

}else{

m1 <- suppressMessages(suppressWarnings(try(lme4::glmer(factor(outcome) ~ Var1 + (1|site), data = df, family=binomial(log), control=glmerControl(optimizer="bobyqa")),silent=T)))

if("try-error" %!in% class(m1)){

res <- exp(cbind(fixef(m1), confint(m1, method = 'Wald')[-1,]))

simulations$rr[i] <- res[2,1]

simulations$lcl[i] <- res[2,2]

simulations$ucl[i] <- res[2,3]

simulations$p_val[i] <- summary(m1)$coefficients[2,4]

simulations$model[i] <- "glmer_nAGQ=0"

}else{

m1 <- suppressMessages(suppressWarnings(try(glm(outcome ~ Var1 + site, data = df, family=quasipoisson),silent=T)))

if("try-error" %!in% class(m1)){

res <- exp(confint.default(m1))

simulations$rr[i] <- exp(coef(m1))[2]

simulations$lcl[i] <- res[2,1]

simulations$ucl[i] <- res[2,2]

simulations$p_val[i] <- summary(m1)$coefficients[2,4]

simulations$model[i] <- "fixed"

}

}

}

}

# INTERACTION ANALYSIS FOR VAR 2

m1 <- suppressMessages(suppressWarnings(try(lme4::glmer(factor(outcome) ~ Var1 + Var1:Var2 + (1|site), data = df, family=binomial(log)),silent=T)))

if("try-error" %!in% class(m1)){

simulations$p_val_inte_var2[i] <- summary(m1)$coefficients[3,4]

}else{

m1 <- suppressMessages(suppressWarnings(try(lme4::glmer(factor(outcome) ~ Var1 + Var1:Var2 + (1|site), data = df, family=binomial(log), nAGQ = 0),silent=T)))

if("try-error" %!in% class(m1)){

simulations$p_val_inte_var2[i] <- summary(m1)$coefficients[3,4]

}else{

m1 <- suppressMessages(suppressWarnings(try(lme4::glmer(factor(outcome) ~ Var1 + Var1:Var2 + (1|site), data = df, family=binomial(log), control=glmerControl(optimizer="bobyqa")),silent=T)))

if("try-error" %!in% class(m1)){

simulations$p_val_inte_var2[i] <- summary(m1)$coefficients[3,4]

}else{

m1 <- suppressMessages(suppressWarnings(try(glm(outcome ~ Var1 + site + Var1:Var2, data = df, family=quasipoisson),silent=T)))

if("try-error" %!in% class(m1)){

tmp <- summary(m1)$coefficients

simulations$p_val_inte_var2[i] <- tmp[nrow(tmp),4]

}

}

}

}

# INTERACTION ANALYSIS FOR VAR 3

m1 <- suppressMessages(suppressWarnings(try(lme4::glmer(factor(outcome) ~ Var1 + Var1:Var3 + (1|site), data = df, family=binomial(log)),silent=T)))

if("try-error" %!in% class(m1)){

simulations$p_val_inte_var3[i] <- summary(m1)$coefficients[3,4]

}else{

m1 <- suppressMessages(suppressWarnings(try(lme4::glmer(factor(outcome) ~ Var1 + Var1:Var3 + (1|site), data = df, family=binomial(log), nAGQ = 0),silent=T)))

if("try-error" %!in% class(m1)){

simulations$p_val_inte_var3[i] <- summary(m1)$coefficients[3,4]

}else{

m1 <- suppressMessages(suppressWarnings(try(lme4::glmer(factor(outcome) ~ Var1 + Var1:Var3 + (1|site), data = df, family=binomial(log), control=glmerControl(optimizer="bobyqa")),silent=T)))

if("try-error" %!in% class(m1)){

simulations$p_val_inte_var2[i] <- summary(m1)$coefficients[3,4]

}else{

m1 <- suppressMessages(suppressWarnings(try(glm(outcome ~ Var1 + site + Var1:Var3, data = df, family=quasipoisson),silent=T)))

if("try-error" %!in% class(m1)){

tmp <- summary(m1)$coefficients

simulations$p_val_inte_var3[i] <- tmp[nrow(tmp),4]

}

}

}

}

}
